# Multi-omics Analysis of Ferroptosis Regulation Patterns and Characterization of Tumor Microenvironment in Patients with Oral Squamous Cell Carcinoma

**DOI:** 10.7150/ijbs.61441

**Published:** 2021-08-12

**Authors:** Wenchao Gu, Mai Kim, Lei Wang, Zongcheng Yang, Takahito Nakajima, Yoshito Tsushima

**Affiliations:** 1 Department of Diagnostic Radiology and Nuclear Medicine, Gunma University Graduate School of Medicine, Maebashi, Japan.; 2 Department of Oral and Maxillofacial Surgery, and Plastic Surgery, Gunma University Graduate School of Medicine, Maebashi, Japan.; 3 Department of Pathology, Fudan University Shanghai Cancer Center, Shanghai, 200032, China.; 4 Department of Oncology, Shanghai Medical College, Fudan University, Shanghai, 200032, China.; 5 Department of Implantology, School and Hospital of Stomatology, Cheeloo College of Medicine, Shandong University & Shandong Key Laboratory of Oral Tissue Regeneration & Shandong Engineering Laboratory for Dental Materials and Oral Tissue Regeneration, Jinan, Shandong, People's Republic of China.; 6 Department of Diagnostic and Interventional Radiology, University of Tsukuba, Ibaraki, Japan.

**Keywords:** Ferroptosis, Oral squamous cell carcinoma, Prognosis, Tumor microenvironment, Immunotherapy

## Abstract

Ferroptosis is a newly recognized mechanism of regulated cell death. It was reported to be highly associated with immune therapy and chemotherapy. However, its mechanism of regulation in the tumor microenvironment (TME) and influence on oral squamous cell carcinoma (OSCC) therapy are unknown. We identified a ferroptosis-specific gene-expression signature, an FPscore, developed by a principal component analysis (PCA) algorithm to evaluate the ferroptosis regulation patterns of individual tumor. Multi-omics analysis of ferroptosis regulation patterns was conducted. Three distinct ferroptosis regulation subtypes, which linked to outcomes and the clinical relevance of each patient, were established. A high FPscore of patients with OSCC was associated with a favorable prognosis, a ferroptosis-related immune-activation phenotype, potential sensitivities to the chemotherapy and immunotherapy. Importantly, a high FPscore correlated with a low gene copy number burden and high immune checkpoint expressions. We validated the prognostic value of the FPscore using independent immunotherapy and pan-cancer cohorts. Comprehensive evaluation of individual tumors with distinct ferroptosis regulation patterns provides new mechanistic insights, which may be clinically relevant for the application of combination therapies in OSCC.

## Introduction

Cell death, which occurs in all organisms, is an important aspect of development. In particular, certain mechanisms of cell death depend on a specific molecular machinery, which may be pharmacologically or genetically controlled. Ferroptosis, discovered in 2012, is an iron-dependent, nonapoptotic mechanism of cell death, characterized by iron-dependence, and excess levels of reactive oxygen species and lipid peroxidation 1. The cell morphology and function of ferroptosis clearly differs from those of necrosis, apoptosis, and autophagy 2, 3. Further, ferroptosis is closely associated with numerous diseases, such as cancers, neuropathies, as well as kidney injury 4.

Cancer cells require increased levels of iron and lipid metabolism compared with those of normal cells to promote development. Thus, cancer cells are more sensitive to ferroptosis 5, 6. Further, inducers of ferroptosis, such as erastin and RLS3, show strong potential for suppressing the growth of colorectal cancer, clear cell renal cell carcinoma, and melanoma 7, 8.

Head and neck squamous cell carcinoma (HNSCC) represent the sixth most common cancer worldwide with at least 350,000 deaths every year 9, 10. Oral squamous cell carcinoma (OSCC), the most frequently occurring subtype of HNSCC, is characterized by high morbidity and mortality 11. Most patients with OSCC are diagnosed only when the disease is advanced. However, current treatments for OSCC, such as chemoradiotherapy and surgery, have not significantly improved the survival rate 11.

The increased recognition of the complexity and importance of the tumor microenvironment (TME) has inspired numerous investigations of the regulation of the TME in tumor metastasis 12, 13. Moreover, evidence continues to indicate that the expression of ferroptosis regulators is highly associated with the immune response and the TME 14. For example, immune checkpoint inhibitor (ICI) therapies have revolutionized the treatment of certain cancers 15, 16. In 2016, the US Food and Drug Administration approved PD-L1 and PD-1 for treatment of HNSCC 17. In 2021, PD-1 has been used as a first-line therapy for patients with advanced OSCC according to the NCCN guidelines (https://www.nccn.org/home).

Furthermore, ICI treatment enhances CD8+ T cell-mediated ferroptosis in melanoma and ovarian cancer 18. Unfortunately, insufficient information is available that describes the overall characteristic infiltration of the TME mediated by interconnected functions of multiple regulators of ferroptosis, and some studies concentrate on one type of cell or some regulators 19-21. Therefore, characterizing the role of ferroptosis in the cellular infiltration of the TME will enhance our understanding of the antitumor response of components of the TME and improve immunotherapy strategies.

In this study, the first identification of ferroptosis regulation patterns and the characterization of the TME in patients with OSCC was accomplished using samples from the Gene-Expression Omnibus (GEO) and The Cancer Genome Atlas (TCGA) database. This study identified three TME-relevant phenotypes as follows: immune-inflamed, immune-desert, and immune-excluded 22. Moreover, we discovered that the immune-inflamed phenotype is a ferroptosis phenotype.

Moreover, we developed the FPscore to comprehensively evaluate individual tumors with each of the above ferroptosis patterns. The findings reveal that the FPscore shows promise as a prognostic biomarker for patients undergoing chemotherapy and immunotherapy. Therefore, we believe that this information will provide new insights into the regulation of ferroptosis in association with the TME and serve as a platform for developing new strategies to implement optimal combination therapy of cancer.

## Materials and methods

### Data acquisition

Data for patients with OSCC were downloaded from the GEO and TCGA, which included the original “CEL” files of seven GEO data (GSE41613, GSE42743, GSE9844, GSE30784, GSE74530, GSE78060, and GSE138206; n = 510) and the level 3 gene-expression data (counts) of the TCGA-HNSCC cohort. Background adjustment and quantile normalization were performed for GEO data using the “affy” package. We selected 292 samples with OSCC and 30 normal control samples from TCGA-HNSCC data according to the previously described position of the tumor 23. To establish TCGA-OSCC datasets, RNA sequencing, somatic mutation, and copy number aberrations data were downloaded from Genomic Data Commons (GDC, http://portal.gdc.cancer.gov/) and the Broad Institute (https://www.broadinstitute.org/). The original counts data were transformed into transcripts per kilobase million (TPM) values, because the TPM values were more similar to those of microarrays. Batch effects were corrected using the “Remove Batch Effects” algorithm to eliminate nonbiological technical biases for the seven datasets. The GEO + TCGA cohort represented the combination of TCGA-OSCC, GSE41613, and GSE42743 cohorts where every sample contained overall survival (OS) information. The accession numbers, platforms, and other details of datasets are summarized in **Table S1**.

### Consensus clustering of ferroptosis regulation patterns

Ferroptosis regulators were downloaded from http://www.zhounan.org/ferrdb/. After matching with our datasets, we included 232 ferroptosis regulatory genes (**Table S2**). Based on these genes and the GEO + TCGA cohort, data were selected to perform unsupervised clustering analysis using the “ConsensusClusterPlus” R package. Further, this consensus clustering algorithm was used to identify the number of clusters, and the analysis included 1000 iterations to ensure the stability of the classification 24.

### Gene set variation analysis and functional analysis

The “ClusterProfiler” R package was used for the analyses of the Gene Ontology (GO) and Kyoto Encyclopedia of Genes and Genomes (KEGG) databases. A false discovery rate (FDR) < 0.05 served as the cutoff to select the pathways that were significantly enriched. Non-parametric and unsupervised Gene Set Variation Analysis (GSVA) analysis was performed using the “GSVA” R package and gene sets of the MSigDB database of the Broad Institute. Single-sample gene set enrichment analysis (ssGSEA), including all TME and immune cell signatures, was conducted using the package “IOBR” 25. The enrichment scores were calculated to represent the relative expression in each sample.

### Differentially expressed gene (DEG) analysis and development of the ferroptosis score (FPscore)

To identify genes associated with ferroptosis patterns, DEGs among the ferroptosis clusters were determined using the limma R package. The significant criteria were selected using FDR < 0.01 and absolute fold-change (FC) > 2. To evaluate the classification value of the DEGs, dimension reduction was conducted using the Boruta algorithm 26 and the ferroptosis gene signature was determined. The unsupervised clustering method was performed using the TCGA-OSCC dataset. Patients were divided into different groups according to the clustering of ferroptosis gene signature for further analysis.

PCA was then performed to determine the FPscore using principal components 1 and 2. This approach concentrated on the score of the set comprising the most significantly associated genes and involved scaling down the score of genes that did not track to other members of the set. The FPscore, which is described according to a GGI-like procedure 27, was calculated as follows: FPscore = ∑(PC1_i_-PC2_i_).

### Evaluation of the FPscore of the TCGA cohort

To evaluate the FPscore of TCGA-OSCC data, we first used Kaplan-Meier analysis to determine high and low FPscore subtype via the “survminer” R package. Next, HALLMARK gene set (h.all.v7.2.symbols) enrichment analysis (GSEA) was used to determine the biological signaling pathways associated with two groups using the “clusterProfiler” R package (P < 0.05, FDR < 0.25) 28. To assess the importance of the association of the FPscore with patients' clinical characteristics, univariate and multivariate analyses were performed to establish a Cox proportional hazard regression model. The tumor mutation burden (TMB) of OSCC was calculated using the total number of nonsynonymous mutations per megabase with the “maftool” R package 29. The chi-square test was performed to evaluate the significance of differences of the numbers of mutated genes between FPscore subtypes. GISTIC 2.0 (https://cloud.genepattern.org) was used determine copy number alterations to classify genes that were amplified or deleted 30. The total number of each gene with an arm or focal region, which was deleted or amplified, was calculated as the burden of copy number loss or gain 31.

### Prediction of the immunotherapeutic response and evaluation of drug sensitivity

The immunophenoscore, tumor immune dysfunction and exclusion (TIDE) algorithm, and the subclass mapping (submap) algorithm were used to predict responses to ICI treatment as previously described 32-34. To predict the chemotherapeutic responses of FPscore subtypes, the drug sensitivity data for cancer cell lines was obtained from the Cancer Therapeutics Response Protal (CTRP v2.0, https://portals.broadinstitute.org/ctrp) and the PRISM Repurposing dataset (PRISM, https://depmap.org/portal/prism/). The CTRP and PRISM databases contained 481 and 1448 compounds, respectively. We evaluated the most widely used drugs to treat OSCC (afatinib, aphidicolin, capecitabine, cisplatin, etoposide, fluorouracil, and paclitaxel) as well as the other four inducers of ferroptosis, which included RSL-3, erastin, ML162, and ML210. The lower area under the curve (AUC) of the dose-response curve indicated increased sensitivity to a chemotherapeutic drug 35.

### Connectivity map analysis

To further identify candidate compounds that target FPscore subtypes and to classify potentially beneficial therapeutics, connectivity map analysis (CMap, https://clue.io/) was conducted utilizing genes with the most significant fold-changes (up- and downregulated; absolute FC > 1.5, FDR < 0.05) 36. Compounds with enrichment scores > 0.5 (P < 0.05) were designated potential agents for treating patients with OSCC.

### Influence of the ferroptosis patterns on immunotherapy and TCGA analyses of pan-cancer

We analyzed five independent immunotherapy datasets, which included the melanoma cohort treated with pembrolizumab (anti-PD-1, GSE78220, n = 27) 37, advanced melanoma treated with nivolumab (anti-PD-1, GSE91061, n = 50) 38, melanoma treated with the immunotherapy TCGA-SKCM (n = 70) 39, melanoma treated with ipilimumab and nivolumab or pembrolizumab (anti-PD-1 and anti-CTLA4, Gide. El at cohort, n = 32) 40, and metastatic urothelial cancer treated with atezolizumab (anti-PD-L1, IMvigro210, n = 298) 41. Data were transformed from FPKM to TPM for further analysis. Survival analysis was performed to validate the prognostic value of the FPscore as applied to ICI treatment cohorts. Further, time-dependent receiver operating characteristic (ROC) curves were generated to evaluate the predictive significance of the FPscore. The pan-cancer data of 33 independent TCGA cancer cohorts comprising 9703 tumor samples were acquired using the UCSC Xena browser (http://xena.ucsc.edu/), and the correlations between FPscore and PD-L1, PD-1, CTLA-4, GPX4 in the pan-cancer cohort were evaluated. The prognostic value of the FPscore for predicting the OS was validated through univariate Cox regression analysis and displayed as a forest plot.

### Statistical analysis

Data were analyzed using R software (version 3.63). Comparisons of ≥ 2 groups were conducted using a parametric test (Student *t-*test or ANOVA test) or a nonparametric test (Wilcoxon rank-sum test or Kruskal-Wallis test). Post hoc tests were performed with Benjamini-Hochberg adjustment of P values after a Kruskal-Wallis test to compare each pair of groups using the R package “PMCMR”. Pearson correlation coefficients of the two groups were compared. Additionally, chi-square tests were used to analyze correlations between the ferroptosis gene clusters and OSCC clinicopathological characteristics. Survival analysis was performed using the Kaplan-Meier method, and the significance of differences was evaluated using the log-rank test. Univariate and multivariate analyses were used to establish a Cox proportional hazard regression model and a nomogram model. We generated a time-dependent ROC curve to estimate the power of the nomogram model. The AUC was calculated using the R package “pROC”. Alluvial diagrams show the distribution of clusters. The data in nomograms included the standard error of the mean (SEM). P < 0.05 indicates a significant difference, and ns, *, **, and *** represent not significant (P ≥ 0.05) and significant at the levels P < 0.05, P ≤ 0.01, and P ≤ 0.001.

## Results

### The ferroptosis regulation patterns in OSCC

**Figure 1:**
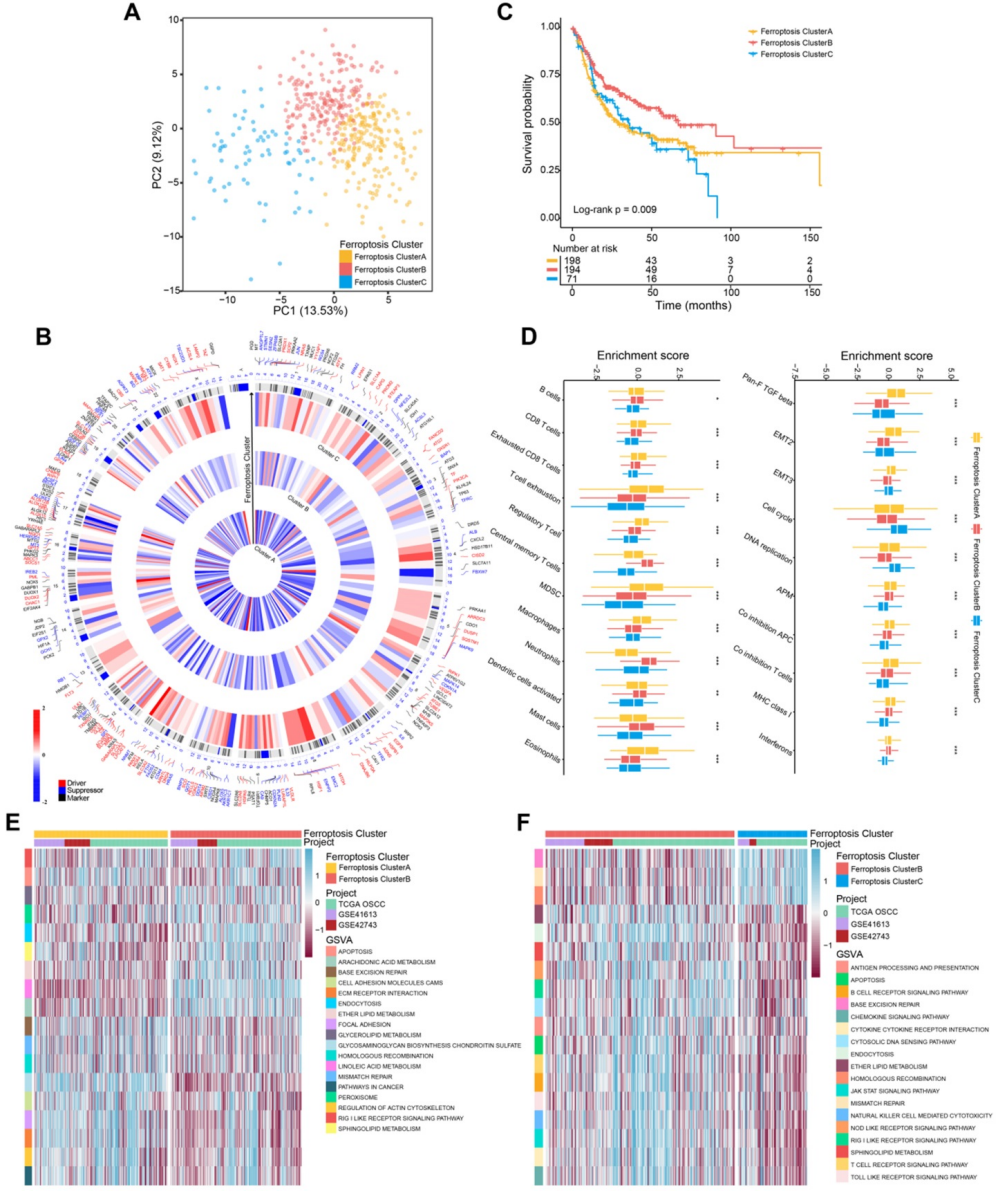
** Ferroptosis regulation patterns and related biological process. (A)** Principal component analysis of 463 (GSE41613, GSE42743, TCGA-OSCC) patients with OSCC. There were significant differences among the transcriptomes of three distinct ferroptosis regulation patterns. **(B)** Integration of circular plots of 463 patients with OSCC. Circular tracks from outside to inside: genome positions according to chromosomal position (black lines, cytobands), ferroptosis cluster A, B, or C in each track (red: Driver; blue: Suppressor; black: Marker). **(C)** Survival analyses of patients (n = 463) with these regulation patterns designated ferroptosis cluster A (n = 198), ferroptosis cluster B (n = 194), and ferroptosis cluster C (n = 71). Kaplan-Meier analysis (log-rank p = 0.009) showed significant differences in OS among the three patterns. **(D)** The enrichment score of infiltrating immune cells associated with the three ferroptosis regulation patterns and the TME signatures of the three clusters. **(E,F)** GSVA enrichment analysis showing the different activation states of biological pathways associated with the ferroptosis regulation patterns. Ferroptosis cluster A vs. ferroptosis cluster B and ferroptosis cluster B vs. ferroptosis cluster C. Heat map: blue, activated pathways; red: inhibited pathways. The different cohorts served as sample annotations. P values were evaluated using the Student's t-test and Kruskal-Wallis test (*P < 0.05, **P < 0.01, ***P < 0.001).

According to the results of unsupervised cluster analysis, k = 3 was selected as the optimal ferroptosis cluster number, according to the expression levels of 232 ferroptosis regulators (**Figure S1A-E**;**Table S2**). Using these data, we distinguished ferroptosis regulation patterns as ferroptosis cluster A, ferroptosis cluster B, and ferroptosis cluster C by PCA (**Figure 1A**). The circos plot shows the expression levels of genes encoding ferroptosis regulators in the clusters and the chromosomal positions of their corresponding genes (**Figure 1B**). Kaplan-Meier survival analysis showed that these ferroptosis regulation patterns significantly differed with patients' survival (log-rank test, P = 0.009) (**Figure 1C**).

To identify the biological differences that potentially explain these differences in survival, we investigated immune-cell infiltration and the TME associated with ferroptosis regulation patterns. Among three ferroptosis clusters, all of their immune-cell populations and TME were significantly different. Further, the ferroptosis cluster C subtype, which was associated with the shortest survival, comprised of fewer immune cells; and the ferroptosis cluster A and B subtypes were mainly enriched in immune cells, such as B cells and CD8 T cells (**Figure 1D**;**Table S3**). But the populations of exhausted CD8 T cells, T cell exhaustion, and regulator T cells were significantly elevated in ferroptosis cluster A. The TME results showed marked enrichment of ferroptosis cluster A in TGF-β, the epithelial-mesenchymal transition (EMT), co-inhibition antigen-presenting cells (APC), co-inhibition T cells, and major histocompatibility complex (MHC) Class I. GSVA analysis revealed that ferroptosis cluster A was strongly associated with stromal activation, which included extracellular matrix receptor interaction, focal adhesion molecules, and cell adhesion molecules (**Figure 1E**;**Table S4**). Ferroptosis cluster B was associated with ferroptosis-related metabolism and immune response pathways (**Figure 1E-F**; **Table S4**). Moreover, ferroptosis cluster C was associated with DNA repair pathways (**Figure S1F**;**Table S4**). Together, these findings indicated that ferroptosis cluster B was associated with immune activation and ferroptosis-related activities. Ferroptosis cluster C showed more genomic instability and less immune activation than the other two clusters. In contrast, ferroptosis cluster A showed T cell suppression and activation of stromal cells.

### Identification of genes associated with ferroptosis regulatory subtypes

**Figure 2:**
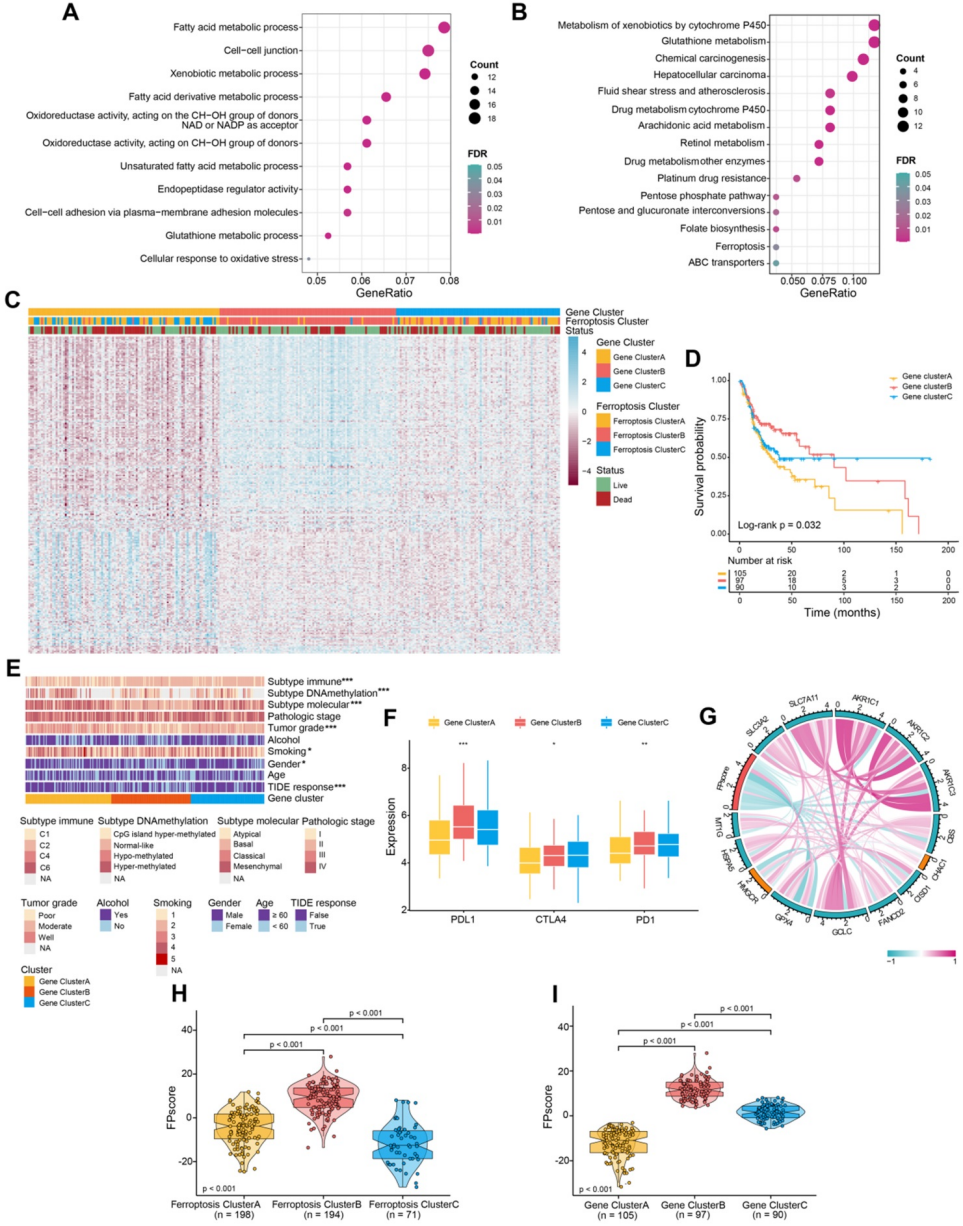
** Identification of ferroptosis gene clusters and development of the FPscore. (A,B)** Functional annotation of DEGs using GO and KEGG enrichment analysis. **(C)** Unsupervised clustering of ferroptosis regulation patterns-related DEGs was used to classify patients into different genomic subtypes in the TCGA-OSCC cohort (ferroptosis gene clusters A, B, and C). The gene clusters, ferroptosis clusters, and patients' clinical characteristics were used as patients' annotations. Blue, high expression of regulators; red, low expression **(D)** The survival of patients in the ferroptosis gene clusters were estimated using the Kaplan-Meier plotter (P = 0.032, log-rank test). **(E)** Correlations between ferroptosis gene clusters and patients' clinicopathological characteristics. **(F)** The distributions of PD-L1, CTLA4, PD-1 expression among the ferroptosis gene clusters. **(G)** Correlation between FPscore and canonical ferroptosis-related genes.** (H,I)** Violin/boxplot showing the distributions of FPscore in ferroptosis clusters A, B, and C and ferroptosis gene clusters A, B, and C. P values were evaluated using the chi-square, Kruskal-Wallis tests as well with Pearson correlation analysis (*P < 0.05, **P < 0.01, ***P < 0.001).

To evaluate the transcriptome differences among ferroptosis regulation patterns, we conducted analyses of DEGs (**Table S5**) together with the Boruta algorithm to minimize dimensions of the ferroptosis gene signature to reduce noise or redundant genes and 245 DEGs were finally obtained as ferroptosis gene signatures (**Table S6**). We next analyzed the TCGA-OSCC cohort (292 patients with OSCC; **Table S7**) to better identify and recognize the biological and clinical variations between these patterns. The summaries of GO and KEGG analyses of the DEGs are shown in **Figure 2A-B,** and **Table S8**. DEGs were significantly enriched in ferroptosis-related metabolism pathways, including fatty acid metabolism and the metabolism of xenobiotics by cytochrome P450 (FDR < 0.05).

Unsupervised clustering was performed according to the expression of 245 DEGs. We identified three genomic clusters in the TCGA-OSCC cohort, namely ferroptosis gene clusters A, B, and C (**Figure S2A-E**). The heatmap illustrated significantly different transcriptome profiles of the 245 DEGs according to genomic cluster (**Figure 2C**). Further, Kaplan-Meier survival analysis revealed important prognostic variations in the TCGA-OSCC cohort associated with the three ferroptosis gene clusters as follows: The ferroptosis gene cluster B correlated with better prognosis, whereas ferroptosis gene clusters A and C were associated with worse outcomes (P = 0.032) (**Figure 2D**). The chi-square test stratified patients into three discrete clusters with distinct ferroptosis gene signature and clinicopathological characteristics. The ferroptosis gene clusters were significantly associated with differences in the immune response according to the TIDE algorithms (P < 0.05). Furthermore, gene cluster B exhibited low tumor grades and mainly the basal molecular subtype, whereas gene clusters A and C showed diverse patterns (**Figure 2E**).

Similarities between the landscape of infiltration of immune cells and TME signatures characteristic of the ferroptosis regulation patterns are shown in **Figure S2F-G,** and**Table S3**. Gene cluster B and C exhibited higher B cells and CD8 T cells than were exhibited by gene cluster A. T cell exhaustion and regulatory T cells were most enriched in gene cluster C. Patients with higher expression levels of PD-L1 as well as expression of immune activation-related genes, such as CD8A, CXCL10, and IFNG, were more abundant in gene cluster B than they were in clusters A and C (**Figure 2F**;**Figure S2H**;**Table S3**). These results were consistent with patients' outcomes and the clinical relevance of different gene clusters, indicating that our classification was reasonable.

### Generation of the FPscore

To apply the ferroptosis regulation patterns to each patient with OSCC according to these ferroptosis gene signatures, we developed the FPscore. The circle plot shows that the FPscore was significantly associated (P < 0.05) with canonical ferroptosis-related genes 42, 43 (**Figure 2G**; **Table S9**). Notably, ferroptosis cluster B and gene cluster B, which had the highest FPscore, were associated with better prognosis compared to other clusters (**Figure 2H**-**I**).

### FPscore is an independent prognostic factor in OSCC

**Figure 3:**
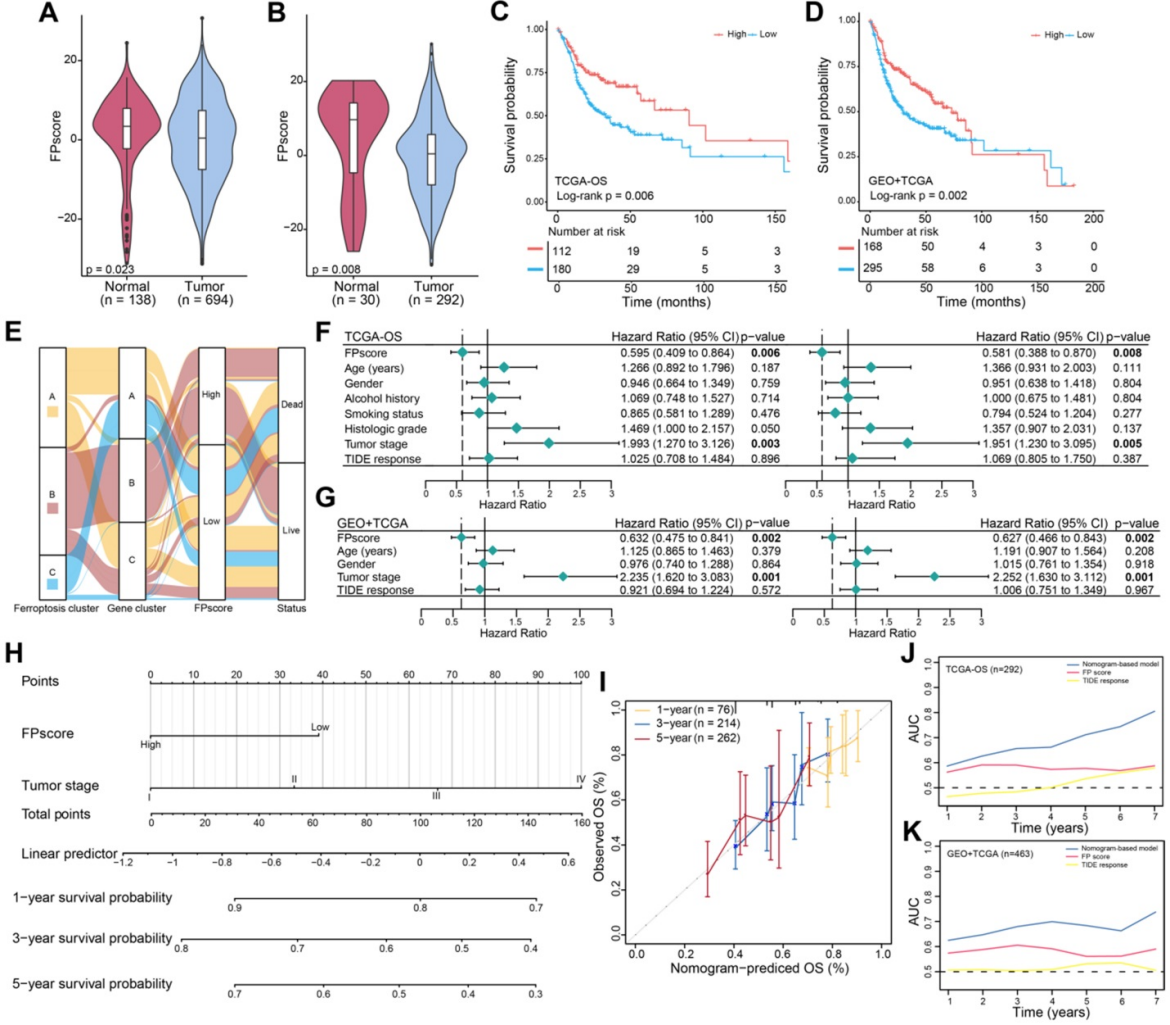
** Ferroptosis score is an independent prognostic factor in OSCC. (A)** Violin/boxplot showing the distributions of FPscore between normal and tumor tissues of patients in cohorts: GSE9844, GSE30784, GSE41613, GSE42743, GSE74530, GSE78060, GSE138206, and TCGA-OSCC.** (B)** Violin/boxplot showing the distributions of FPscore between normal and tumor tissues of patients in TCGA-OSCC cohort.** (C,D)** Kaplan-Meier analysis of OS associated with high and low FPscore subtype in the TCGA-OSCC cohort (log-rank test, P = 0.006) and GEO + TCGA cohort (log-rank test, p = 0.002). **(E)** Alluvial diagram of ferroptosis clusters in groups of ferroptosis gene clusters, FPscore, and survival outcomes. **(F,G)** Forest plot showing univariate and multivariate Cox regression analyses of FPscore associated with age, gender, tumor stage, and TIDE score of two cohorts. **(H)** Nomogram developed using multivariate Cox regression analysis for predicting the OS of patients with OSCC. **(I)** Plots depict the calibration of the model in terms of agreement between predicted and observed OS rates. Model performance is shown, and the 45º slope represents perfect prediction. **(J,K)** AUCs associated with OS of the nomogram-based signatures, FPscore, and TIDE scores of the TCGA-OSCC cohort and GEO + TCGA. P values were tested using the Student's t-test, the Mann-Whitney test, and Kruskal-Wallis test (*P < 0.05, **P < 0.01, ***P < 0.001).

Further, the FPscore was significantly different between normal and tumor samples in all cohorts (seven GEO datasets and TCGA-OSCC cohort, n = 832) and TCGA-OSCC cohorts (n = 322) (**Figure 3A**-**B**). The prognostic value of the FPscore for predicting OS, progression-free interval (PFI), and the disease-specific survival (DSS) of the TCGA-OSCC cohort was evaluated (**Figure 3C**;**Figure S3A**-**B**). These results were confirmed by Kaplan-Meier analyses of the GEO + TCGA, GSE42743, and GSE41613 cohorts (**Figure 3D**;**Figure S3C**-**D**). Based on the OS results in TCCA-OSSC cohort, we divided the OSCC patients into high/low FPscore subtype and the association between ferroptosis-related clusters and status were displayed in **Figure 3E**. These results showed that the high FPscore subtype experienced significantly better outcomes compared with the low FPscore subtype.

The FPscore, which was subsequently evaluated as a continuous variable in univariate and multivariate Cox regression models, was identified as an independent and stable prognostic factor of the TCGA-OSCC and GEO + TCGA cohorts (HR, 0.58; 95% CI, 0.39-0.87; P = 0.008 and HR, 0.63; 95% CI, 0.47-0.84; P = 0.002, respectively) (**Figure 3F-G**). Further, a nomogram that combined the FPscore with the tumor stage was created to provide clinicians with a predictive tool for estimating the 1-, 3-, and 5-year prognoses of patients with OSCC (**Figure 3H**). The calibration plot showed that the nomogram performed well in predicting OS (**Figure 3I**). The C-indices of our nomogram model used to predict OS of the TCGA-OSCC and GEO + TCGA cohorts were 0.61 and 0.60, respectively. Further, the AUC values showed that the FPscore was superior to the TIDE score for predicting OS (**Figure 3J-K**).

### Characteristics of the FPscore of TCGA molecular subtypes and their clinical relevance

**Figure 4:**
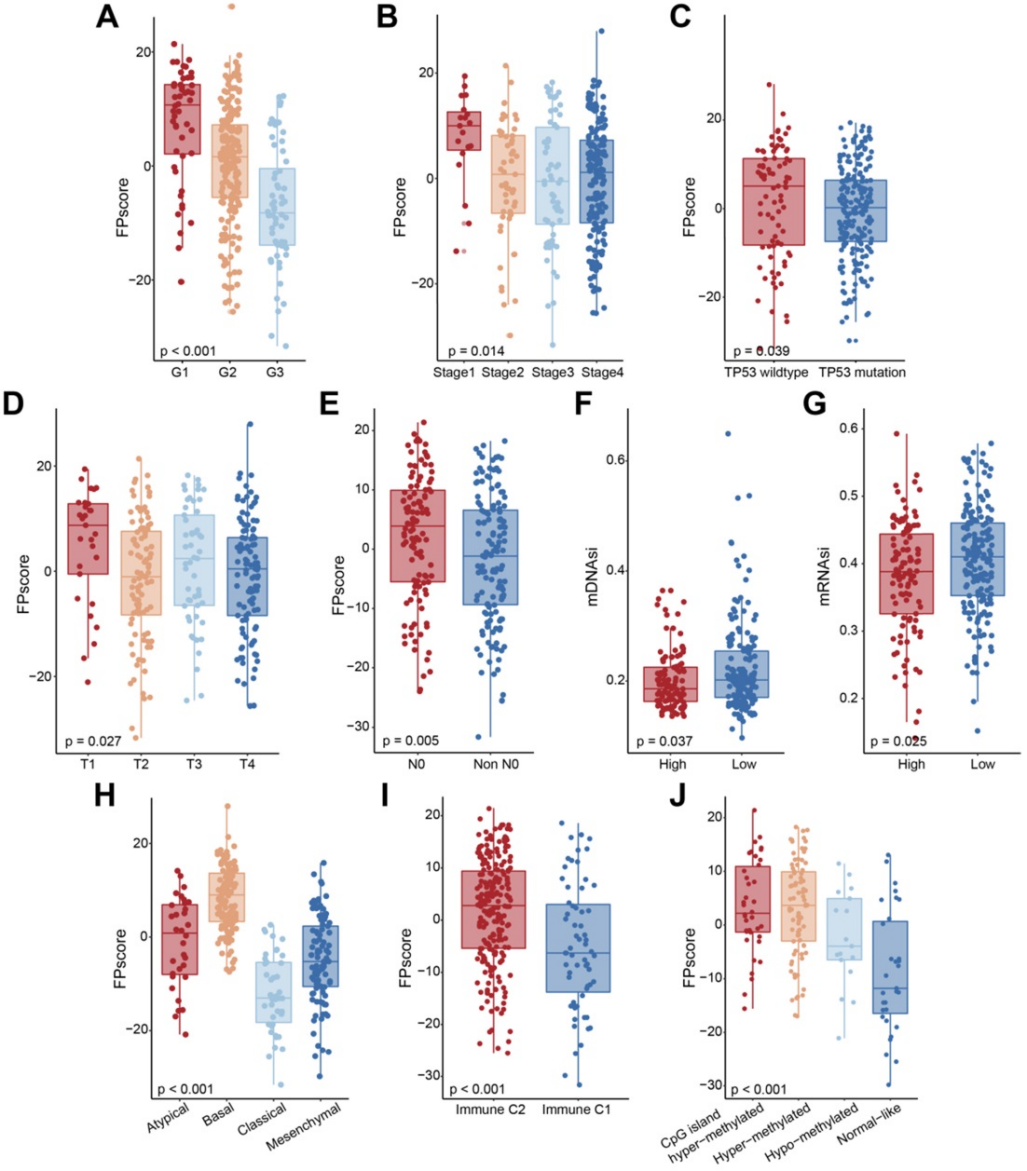
** Characteristics of the FPscore in OSCC molecular subtypes. (A-J)** Boxplots showing the FPscore distributions according to grade, tumor stage, TP53 mutation, and molecular subtype. P values were tested using the Student's t-test, Mann-Whitney test, and Kruskal-Wallis test (*P < 0.05, **P < 0.01, ***P < 0.001).

When we investigated the relationship between the FPscore and clinical characteristics, we found that a high FPscore was significantly associated with low tumor grade, early tumor stage, wild-type TP53, T and N stage, and low mDNAsi and mRNAsi (P < 0.05) (**Figure 4A-G**; **Table S3**). Further, the distributions of FPscore were significantly different among the molecular subtypes (**Figure 4H-J**;**Table S3**).

**Figure 5:**
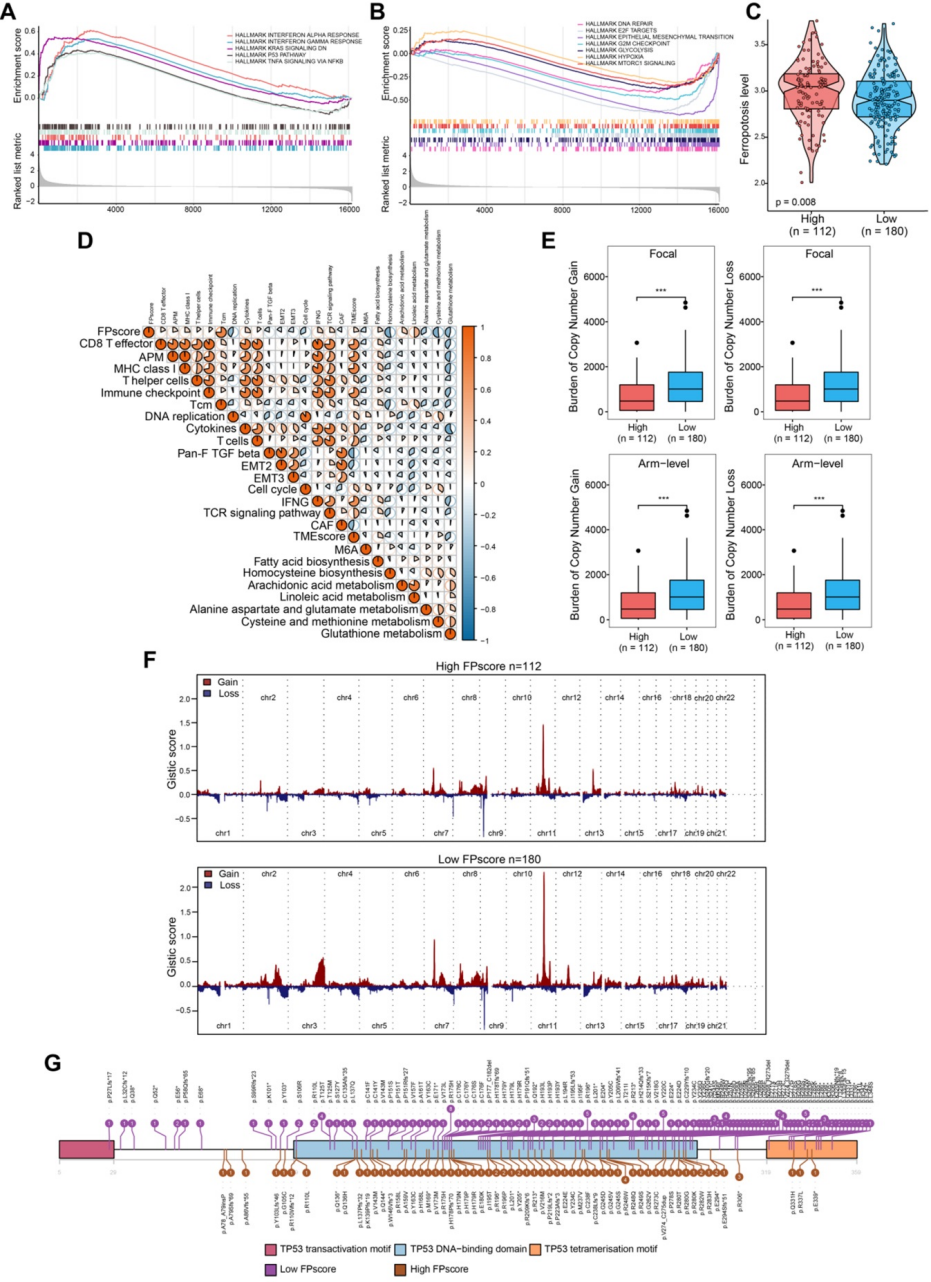
** The biological pathways and copy number burdens of FPscore subtypes. (A)** GSEA enrichment plots showing the associations of the high FPscore subtype with interferon response, KRAS signaling down, P53 pathway, and TNF-alpha signaling. **(B)** GSEA enrichment plots showing the associations of the low FPscore subtype with active in DNA repair, E2F targets, EMT, G2M checkpoint, glycolysis, hypoxia, and mTORC1 signaling pathway. **(C)** Violin/boxplot showing the ferroptosis levels of the FPscore subtypes.** (D)** Correlations between the FPscore associated with the immune-activation signature, TME, and ferroptosis-related metabolism. A negative correlation is shown in blue and a positive correlation in red.** (E)** Distribution of focal and broad copy number aberrations between the FPscore subtypes.** (F)** Copy number profiles of the low and high FPscore subtype, with gains in red and losses in blue. Gene segments are ordered according to their chromosomal locations.** (G)** Lollipop plots of mutations in TP53, upper side (purple) represents the low FPscore subtype, lower side (brown) represent the high FPscore subtype. P values were tested using the Student's t-test, the Mann-Whitney test, and Pearson correlation analyses (*P < 0.05, **P < 0.01, ***P < 0.001).

To further investigate the biological differences between FPscore subtypes, we performed GSEA analysis. The results showed that TP53 pathway, tumor necrosis factor alpha (TNF-alpha signaling, Kras signaling decreasing, and responses to interferon-gamma were activated in the high FPscore subtype. In contrast, the EMT, DNA repair pathway, hypoxia, and mTORC1 were activated in the low FPscore subtype (**Figure 5A-B**). Further, the ferroptosis level was significantly higher in the high FPscore subtype (P < 0.05) (**Figure 5C**).

A correlation matrix heat map showed that the FPscore was significantly and positively associated with the immune-activation signature, TMEscore, fatty acid biosynthesis, and arachidonic acid metabolism; although there were significant negative correlations with stroma, EMT, the m6A signature and glutathione metabolism (P < 0.05) (**Figure 5D**; **Table S10**). Together, these results support the conclusion that the FPscore achieved clinical prognostic value and that the TME was strongly associated with ferroptosis. Moreover, the FPscore accurately reflected the ferroptosis regulation patterns in patients with OSCC.

### Comparison of copy number aberrations and somatic mutations using the FPscore

Changes of gene copy numbers are associated with ICI. We therefore determined the copy number aberrations between the FPscore subtypes. We found that the high FPscore subtype showed a significant lower focal-level gain/loss burden and a lower arm-level gain/loss burden compared with those of the low FPscore subtype (P < 0.05) (**Figure 5E**). The distributions of G-scores among all chromosomes of these subtypes (**Figure 5F**) showed difference in copy number aberrations. In contrast, there was no significant difference in TMB associated with the FPscore subtypes (P = 0.130) (**Figure S4A**). The number of each somatic mutation type in FPscore subtypes was showed in **Figure S4B**. The oncoplot of somatic mutations showed the top 20 most frequently mutated genes (**Figure S4C-D**). The chi-square test revealed that the high FPscore subtype was associated with a significantly low TP53 mutation burden compared with the low FPscore subtype (**Table S11**). A lollipop plot showed similar results and provided details about the positions of the TP53 mutations between the FPscore subtypes (**Figure 5G**).

### Predicting responses to immune therapy and chemotherapy

**Figure 6:**
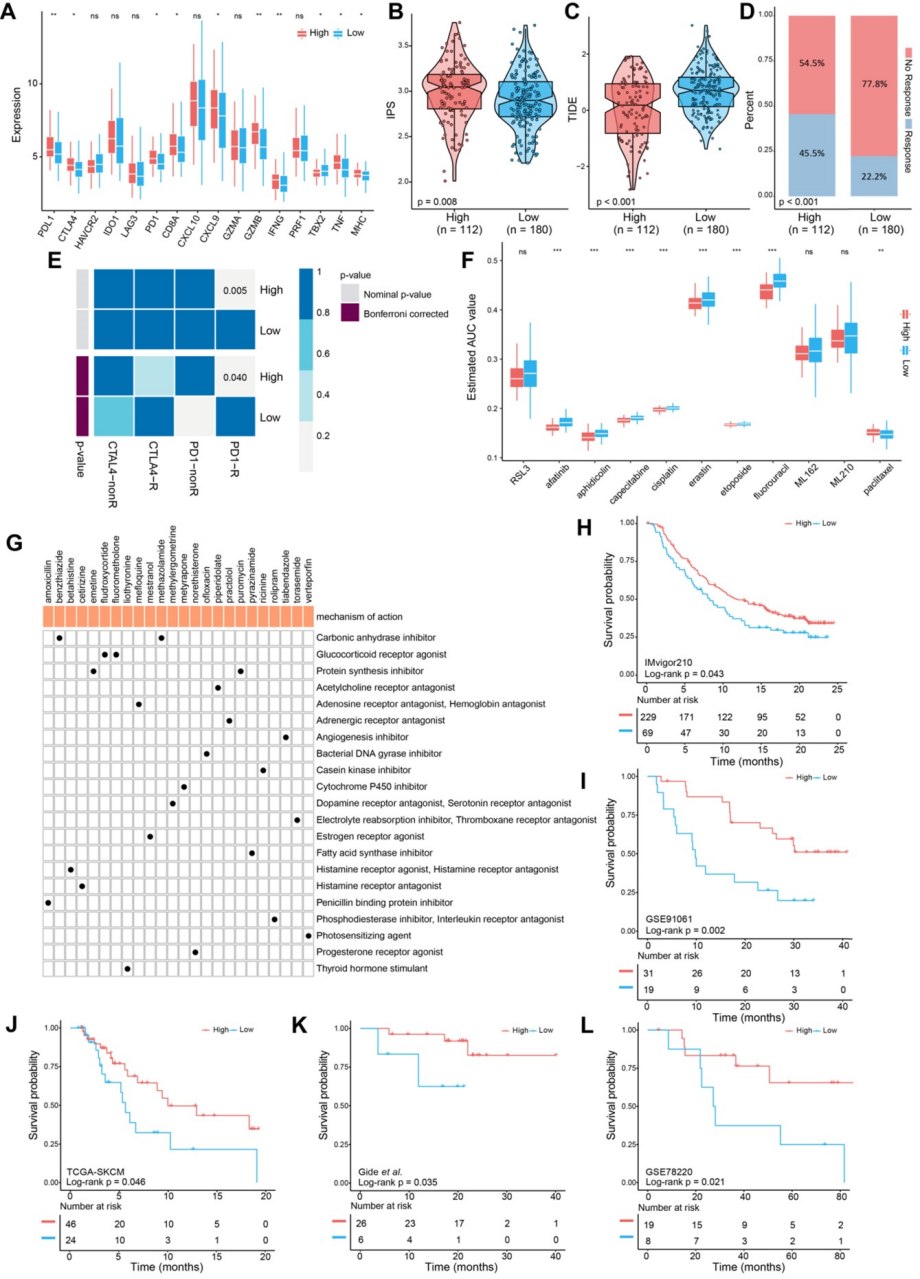
** FPscore subtypes related to the response to immunotherapy and the response to chemotherapy. (A)** Immune-checkpoint-relevant and immune-activation-relevant genes expressed in high and low FPscore subtype.** (B,C)** Violin/boxplot showing significant differences between FPscore subtypes associated with the immunophenoscore and TIDE scores. **(D)** Rates of clinical responses to immunotherapies in the high and low FPscore subtype in the TCGA-OSCC cohort according to TIDE scores.** (E)** Submap analysis indicated that the high FPscore subtype was more sensitive to anti-PD-1 treatment. **(F)** Boxplot of differential drug responses of 11 compounds associated with the FPscore subtype. Lower AUC values on the y-axis of boxplots indicate greater drug sensitivity. **(G)** Heat map showing each compound (perturbagen) from the CMap that shares a mechanism of action (rows) and sorted by descending number of compounds with shared mechanisms of action. **(H)** Kaplan-Meier analyses of OS of high and low FPscore patient groups in the IMvigor210 cohort (log-rank test, P = 0.043), **(I)** the GSE91061 cohort (log-rank test, P= 0.002),** (J)** the TCGA-SKCM cohort (log-rank test, P = 0.046),** (K)** the Gide et al. cohort (log-rank test, P = 0.035), and **(L)** the GSE78220 cohort (log-rank test, P = 0.021). P values were tested using the Student's t-test and the Mann-Whitney test (ns: not significant, *P < 0.05, **P < 0.01, ***P < 0.001).

To determine whether the FPscore predicted the response of ICI treatment in OSCC, we evaluated the expression of immune checkpoint and immune-activation-related genes. There was a significant increase in the high FPscore subtypes including PD-L1, CTLA4, PD-1, IFNG, and MHC (P < 0.05) (**Figure 6A**). Next, we used the immunophenoscore scoring system and TIDE algorithm to evaluate the potential therapeutic effectiveness of immunotherapy. immunophenoscore was found significantly elevated in high FPscore subtype (P < 0.05) (**Figure 6B**). A greater propensity for immune evasion was demonstrated by the higher TIDE predictor score, indicating that patients may not benefit from ICI therapy. Interestingly, the high FPscore subtype had a significantly lower TIDE score compared with that of the low FPscore subtype (P < 0.05) (**Figure 6C**). We next investigated whether the higher FPscore subtype correlated with an objective response to ICI therapy though TIDE (P < 0.001) (**Figure 6D**). The submap result showed that the high FPscore subtype may respond to PD-1 treatment (P < 0.05) (**Figure 6E**). These results provided evidence that the ferroptosis regulation patterns play a key role in mediating the immune response in OSCC.

Chemotherapy is frequently administered to patients with OSCC, although drug resistance is a major problem that hinders treatment. We therefore applied CTRP and PRISM data to delineate drug resistance and sensitivity associated with the FPscore subtype. A high FPscore was associated with sensitivity to chemotherapeutic drugs, and a low FPscore subtype was significantly associated with drug resistance (P < 0.05) (**Figure 6F**). Moreover, we found that the sensitivity of erastin, an inducer of ferroptosis, significantly correlated with the high FPscore subtype. These findings indicated that a patient with a high FPscore may achieve a good response to chemotherapy.

Further, the CMap mode of action analysis identified 21 pathways shared by the 24 compounds. (**Figure 6G**;**Table S12**). To decipher the prognostic value of the FPscore for immunotherapy, we performed Kaplan-Meier analyses of five independent ICI treatment cohorts. Each cohort showed that a high FPscore subtype was associated with better prognosis compared with that of a low FPscore (**Figure 6H-L**) and time-dependent ROC curves validated the accuracy of the FPscore (**Figure S5A-E**). These results suggested that the FPscore has prognostic value for ICI therapy administered patients.

### Analysis of pan-cancer data

**Figure 7:**
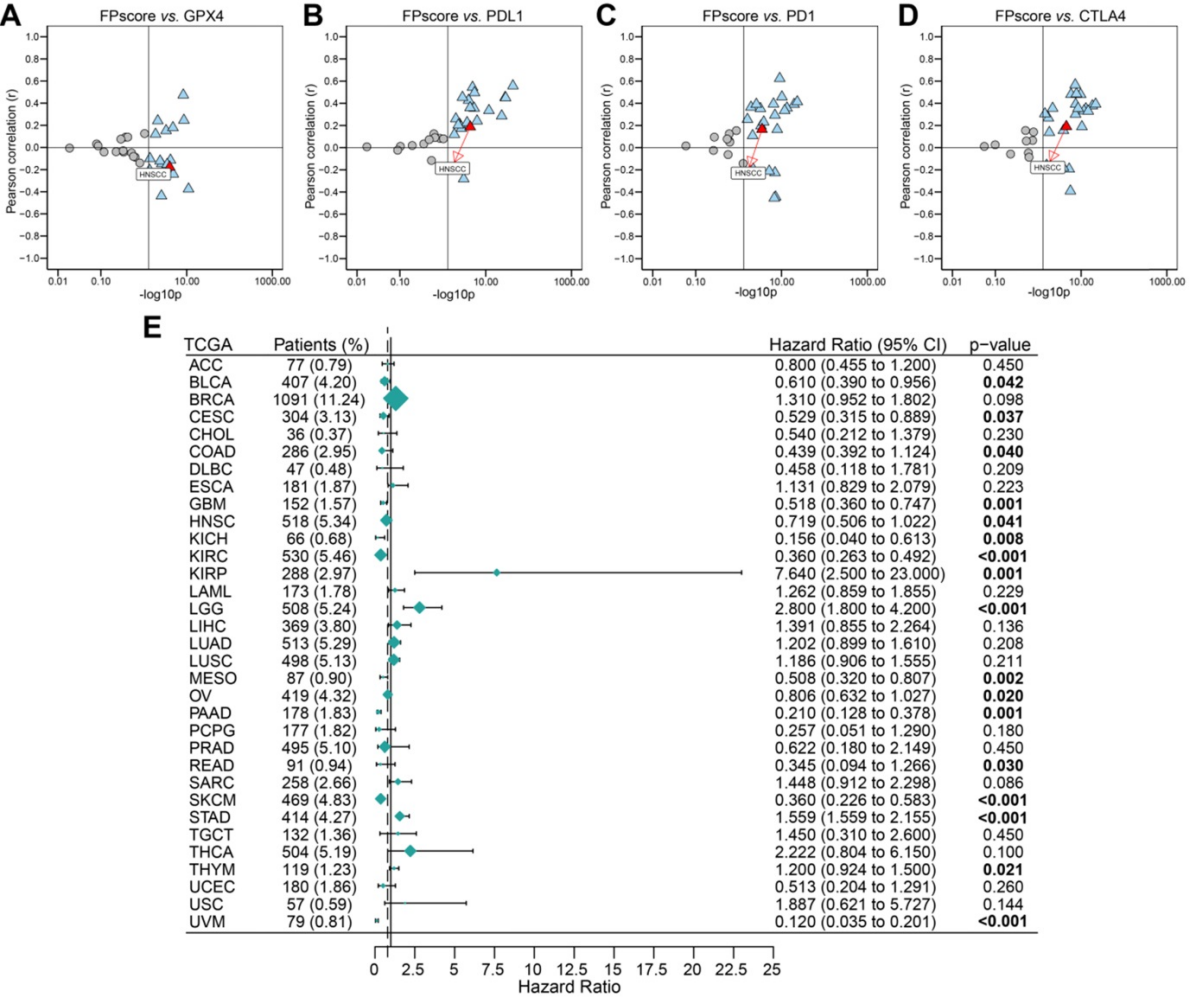
** Evaluation of the utility of the FPscore in pan-cancers. (A-D)** Dot plot showing the correlation between FPscore and GPX4, PD-L1, PD-1, and CTLA4 expression in pan-cancer. The triangle and gray circle represent P > 0.05 and P < 0.05, respectively. **(E)** Univariate Cox regression analyses of the prognostic value of the FPscore in different cancers. The length of the horizontal line represents the 95% CI for each group. The vertical dotted line represents HR = 1. HR < 1.0 indicates that an elevated FPscore is a favorable prognostic biomarker. The numbers of patients are displayed.

To systematically analyze the significance of the FPscore in pan-cancer, we evaluated the expression of the immune check-points and GPX4. The FPscore was significantly associated with GPX4, PD-L1, PD-1, and CTLA4 in many cancers, including HNSCC (**Figure 7A-D**;**Table S13**). A high FPscore was identified as a favorable prognostic biomarker for 13 independent TCGA cohorts, some of which comprised “hot tumors” with high immunogenicity, such as head and neck cancer, colon cancer, kidney cancer, urothelial cancer, and cervical cancer (**Figure 7E**).

## Discussion

**Figure 8:**
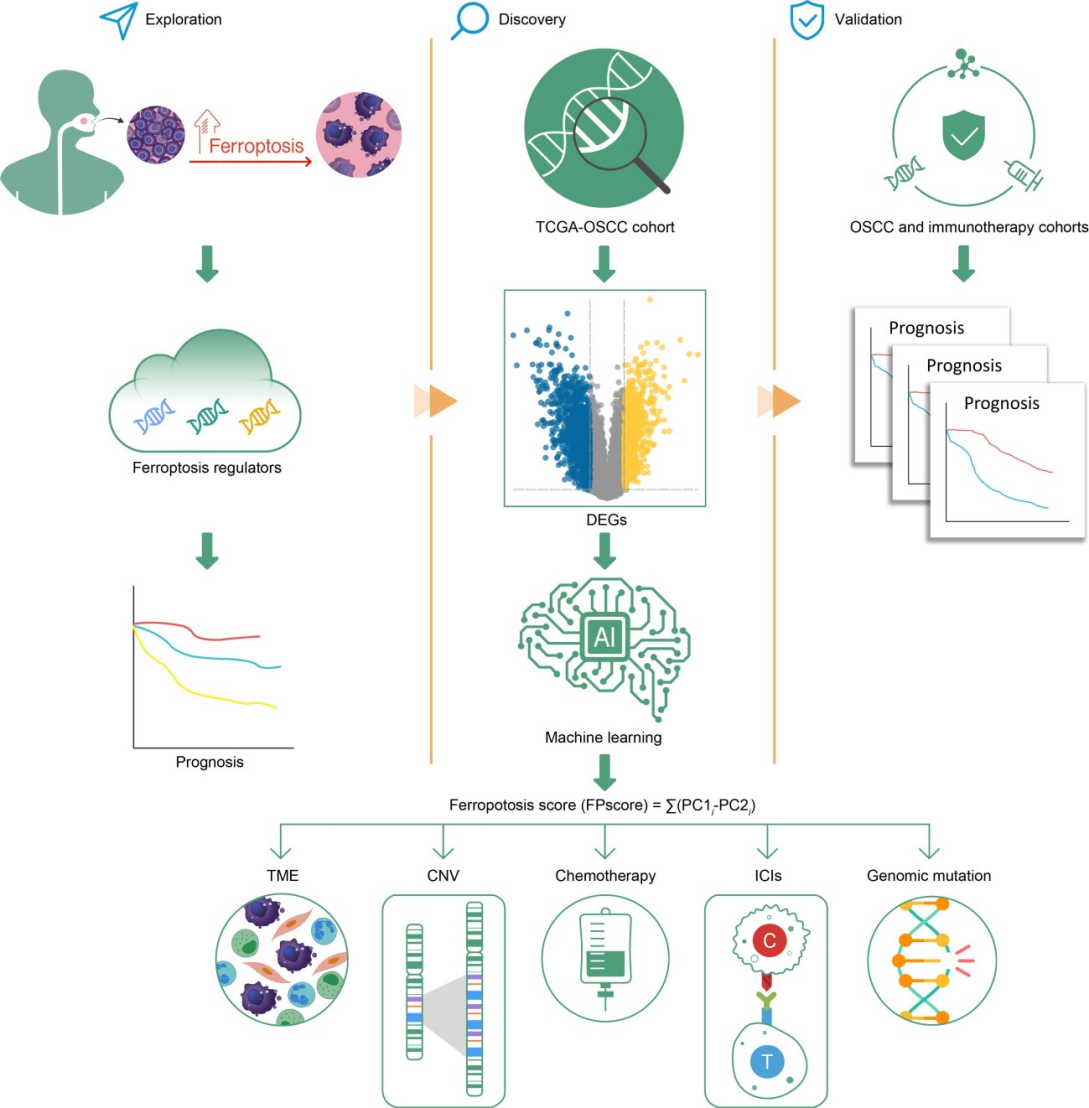
Graphical abstract.

Here we established three distinct ferroptosis regulation patterns, defined by immune phenotypes, which were associated with various levels of anticancer immunity and the scheme of our study is shown in **Figure 8**. This study depicts the identification of ferroptosis cluster A, associated with the immune-excluded phenotype, which was characterized by the stromal activation. Ferroptosis cluster B was characterized by immune-activation and metabolism, which represents an immune-inflamed and ferroptosis phenotype. The ferroptosis cluster C was characterized by less infiltration with immune cells, represented as the immune-desert phenotype (**Figure 1D-F**).

Previous studies showed that stromal activation prevented the infiltration of immune cells in tumor by retaining them in the stroma, which were consequently blocked from penetrating into tumors. And the activation of stromal cells was considered to suppress T cell activation 22, 44. Consistent with these results, ferroptosis cluster A was associated with poor prognosis despite having abundant immune cells. Notably, we identified ferroptosis cluster B, which was associated with better prognosis, activated T cell abundance, and high ferroptosis activity. We believe that it is reasonable to conclude therefore that the activation of T cells enhances ferroptosis and contributes to its strong antitumor effect. For example, ferroptosis is consistent with T cell-mediated cancer immunity 18.

We showed here that the DEGs of distinct ferroptosis patterns were greatly overrepresented in biological pathways involved in ferroptosis-related metabolism, indicating that DEGs represented gene signature linked to ferroptosis phenotypes (**Figure 2A-B**). Similar to the ferroptosis clustering data, three subtypes of transcriptomic profiles were significantly associated with survival outcomes; and the ferroptosis gene signature was established and designated gene clusters A, B, and C. These gene clusters were each significantly associated with a specific clinical subtype (**Figure 2C-D**).

We therefore sought to quantify the ferroptosis trends of individual tumors according to the specific heterogeneity of alterations of ferroptosis. Therefore, the FPscore was developed to identify the ferroptosis patterns of an individual patient. As a result, we found that the FPscore was significantly associated with the expression of canonical ferroptosis-related genes and was consistent with the ferroptosis patterns (**Figure 2G**). And the immune-inflamed and ferroptosis phenotype had a higher FPscore compared with those of the immune-desert and immune-excluded phenotypes (**Figure 2H-I**). These findings indicate that the FPscore will serve as an accurate and robust tool for detailed evaluation of the patterns of alterations of ferroptosis in individual tumors, which may be useful for further evaluation of the patterns of TME, such as tumor immune phenotypes.

Therefore, we conducted a comprehensive evaluation of the FPscore. In clincal practices, Cox regression analysis indicated that the FPscore was an independent clinical predictive factor of the prognosis of OSCC (**Figure 3F-G**). Further, a nomogram model comprising the FPscore and tumor stage achieved good prognostic predictive performance when applied to patients with OSCC (**Figure 3H-K**). However, from a clinical perspective, a large-cohort validation is needed. These results convincingly demonstrate that the nomogram model may provide clinicians with robust prognostic biomarkers for managing patients with OSCC. We further demonstrated that the FPscore was related to clinicopathological characteristics. FPscore was found to be elevated in patients with low grade, early stage, immune C2 subtype, low mRNAsi/mDNAsi, T1 stage, and no lymph node metastasis, which always represents a better prognosis (**Figure 4A-J**). Immune subtype C1 was reported to be highly enriched in angiogenesis and C2 subtype represented the strong CD8 signal and antitumor immune response 45. Moreover, the high value of mRNAsi/mDNAsi in tumors has been identified to reflect the dedifferentiation and metastasis of tumors 46. Taken together, these results indicated that the FPscore is associated with tumor progression and TME remodeling.

Through GSEA, our data revealed that a high FPscore was significantly enriched in ferroptosis-positive regulatory pathways and immune-activation signature (**Figure 5A-B**). For example, arachidonic acid metabolites release oxidized lipid mediators, such as 11-HETE and 15-HETE during ferroptosis to recruit immune cells 6. Fatty acid accumulation in cells and the depletion of cysteine and glutathione peroxidase 4 (GPX4) will also induce ferroptosis 45, 46. Therefore, those factors were in concordance with our results that high FPscore subtype had higher ferroptosis levels (**Figure 5C**). These results also explain that the patients with a high FPscore subtype survived longer compared with those with low FPscore subtype.

We were intrigued by our findings that the FPscore positively correlated with the TMEscore and negatively with the m6A signature (**Figure 5D**). Previous studies showed that a lower m6A signature and a higher TMEscore represented the immune-inflamed phenotype. Both showed a predictive advantage of immunotherapy for gastric cancer 24, 47; here, the TIDE algorithm and the immunophenoscore showed that a high FPscore was significantly associated with a better response to ICI treatment (**Figure 6B-D**). Further, PD-L1, PD-1, and CTLA-4 were significantly expressed at high levels by the high FPscore subtype (**Figure 6A**). Specifically, the submap results imply that the high FPscore subtype may respond to PD-1 treatment (**Figure 6E**). The results of the study by Wang et al. are consistent with our findings using a mice model, in which ICIs enhanced T cell-mediated antitumor immunity though the upregulation of IFN-γ and downregulation of the cystine/glutamate antiporter, system Xc- 18. Recently, several clinical studies have reported that gene mutations may reflect the response of immune therapy; in particular, TMB has been identified as an independent factor for predicting ICI treatment 48, 49. However, some studies have also concluded that high-TMB failed to predict ICI treatment; thus, whether TMB could be a biomarker across all tumors is still unclear 50, 51. When we evaluated the TMB of the two ferroptosis subtypes, we found that they were not significantly different (P = 0.13, **Figure S4A**). In contrast, we were further intrigued that copy number aberrations associated with the FPscore subtype (**Figure 5E-F**). A significantly lower copy number burden in the high FPscore subtype compared with that of the low FPscore subtype was demonstrated. Indeed, copy number aberrations make a larger contribution to the immune signature, and the low burden of copy number gain/loss correlates with the response to immunotherapy 16, 52. A recent report demonstrating that the burden of copy number loss did not correlate with the mutational load during immune therapy 16. Notably, the FPscore may predict the response to immunotherapy as an independent factor or even among OSCC patients with the same TMB level. Furthermore, we found that the low FPscore subtype showed more TP53 mutations than the high FPscore subtype; additionally, the TP53 pathway was activated in tumors with a high FPscore (**Figure 4C**;** Figure 5G**). Therefore, these findings indicate that TP53 may regulate ferroptosis in OSCC cells and are consistent with those of recent studies showing that TP53 and the tumor-associated mutant TP53 may play import roles in ferroptosis of cancer cells 53, 54.

Moreover, the high FPscore subtype showed significant sensitivity to chemotherapeutic agents and to erastin (**Figure 6F**), the latter of which induces ferroptosis of cancer cells through the cysteine-glutamate transporter 55. However, other inducers of ferroptosis, such as RLS3, ML162, and ML210, which directly inhibit GPX4 leading to ferroptosis, did not exhibit drug sensitivity in FPscore subtypes. These findings are likely explained by low GPX4 expression in the high FPscore subtype 5, 56. We used CMap to identify other inhibitors that may have antitumor efficacy in OSCC (**Figure 6G**). The above suggest that ferroptosis represents a pathway in cancers cells that can be effectively targeted by therapeutic agents. For example, we validated the prognostic value of the FPscore of five independent ICI treatment cohorts (**Figure 6H-L**), the same results were obtained. That is, patients with a high FPscore experience a better prognosis than those with a low FPscore, suggesting that the FPscore predicts the survival ratio of patients who undergo immunotherapy. Further, our present pan-cancer analysis showed that the FPscore was associated with the immune checkpoints and GPX4 (**Figure 7A-D**), which predicted the OS of patients with “hot tumors.” These findings support our conclusion that the FPscore could be suitable for translation to the clinic.

In short, the high FPscore subtype was defined as the ferroptosis-related immune-activation subtype and was associated with clinicopathological features, various biomarkers including TME, immune checkpoint expression, and copy number aberrations, which indicates that the FPscore is a promising prognostic biomarker related to ICI treatment and chemotherapy in OSCC. However, further studies including single-cell RNA-seq and proteomics data analyses are required to identify the details of the activation of T cells caused by ferroptosis, such as the mechanism of ferroptosis-induced cell death, which releases signals that trigger cytotoxic T cell-mediated adaptive immunity, which is beyond the scope of the current study. Moreover, ferroptosis also occurred in the normal cells and play a role as a double-edged sword in oncogenesis through the release of signaling molecules that inhibit or promote tumor growth and proliferation 57. A critical cutoff value and the role of FPscore in assessing prognosis and the immunotherapy responses of OSCC remain to be accurately determined through prospective studies.

## Conclusions

In conclusion, multi-omics analysis revealed that a high FPscore identified patients with OSCC with ferroptosis-related immune activation, who experienced longer survival and benefited from immunotherapy and chemotherapy in OSCC. These findings contribute toward new insights into the regulation of ferroptosis associated with the TME, which may be clinically relevant for developing combination therapies of OSCC.

## Supplementary Material

Supplementary figures.

Supplementary tables.

## Abbreviations

HNSCC: head and neck squamous cell carcinoma
OSCC: oral squamous cell carcinoma
TME: tumor microenvironment
ICI: immune checkpoint inhibitor
GEO: Gene-Expression Omnibus
TCGA: The Cancer Genome Atlas
OS: overall survival
GO: Geno Ontology
KEGG: Kyoto Encyclopedia of Genes and Genomes
GSVA: unsupervised gene set variation analysis
ssGSEA: single-sample gene set enrichment analysis
DEG: differentially expressed gene
FPscore: ferroptosis score
PCA: principal component analysis
GSEA: gene set enrichment analysis
TIDE: tumor immune dysfunction and exclusion
Submap: subclass mapping
Cmap: connectivity map analysis
