# Supplementary material for: Multi-omics Analysis of Ferroptosis Regulation Patterns and Characterization of Tumor Microenvironment in Patients with Oral Squamous Cell Carcinoma
Source: Int J Biol Sci. 2021 Aug 12;17(13):3476–92. doi: 10.7150/ijbs.61441 (PMC8416738; doi:10.7150/ijbs.61441)
Supplement: Supplementary file 1 — Supplementary figures. [file ijbsv17p3476s1.pdf]

Supplementary figures and figure legends

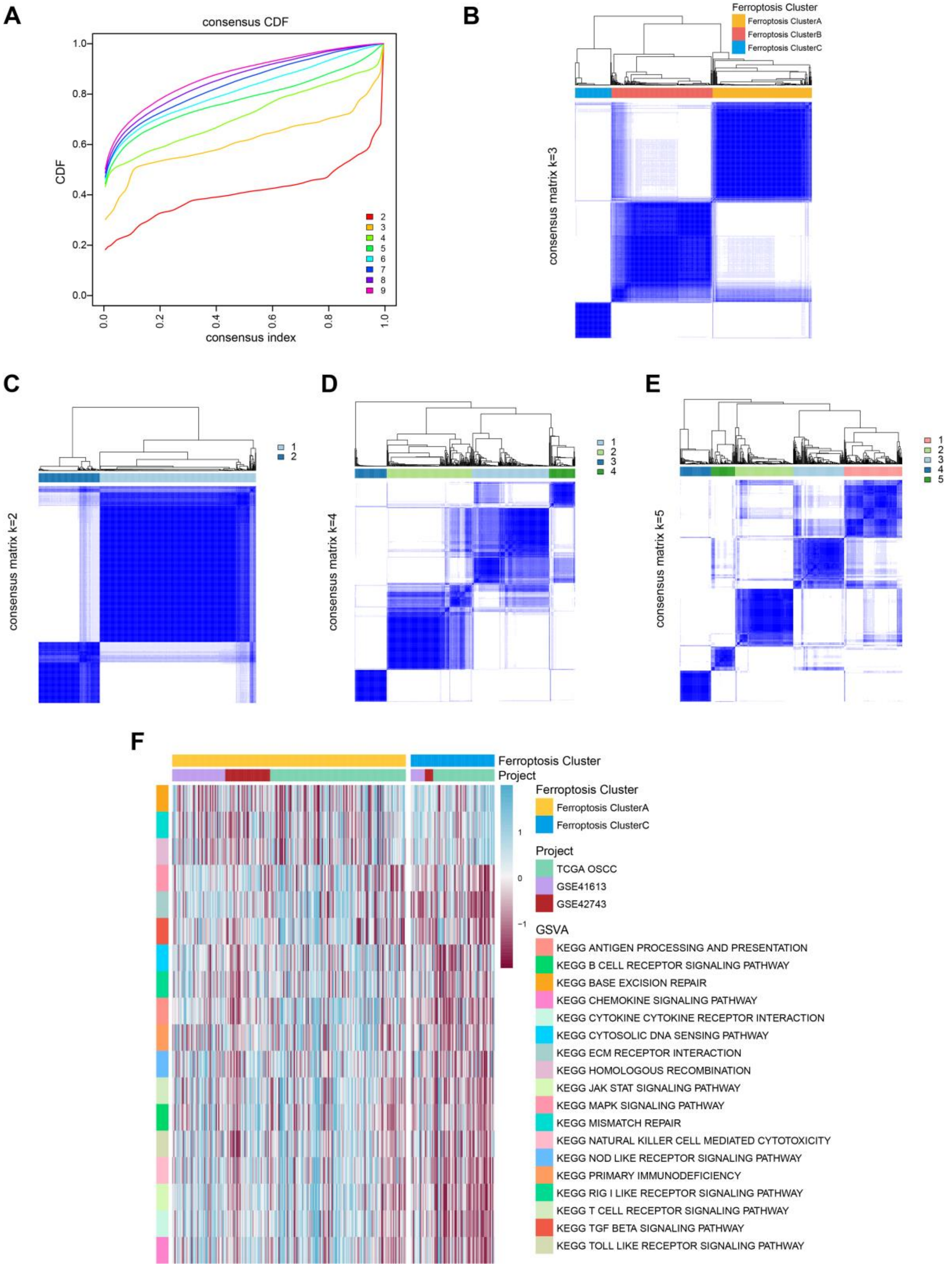

Figure S1 Unsupervised clustering of ferroptosis regulators of patients with OSCC

(A–E) Consensus matrices of 463 patients with OSCC (GSE41613, GSE42743, TCGA-OSCC) (k = 2–5). **(F)** GSVA enrichment analysis showing the different activation states of biological pathways associated with the ferroptosis regulation patterns. Ferroptosis cluster A vs. ferroptosis cluster C. Heat map: blue, activated pathways; red: inhibited pathways. The different cohorts served as sample annotations.

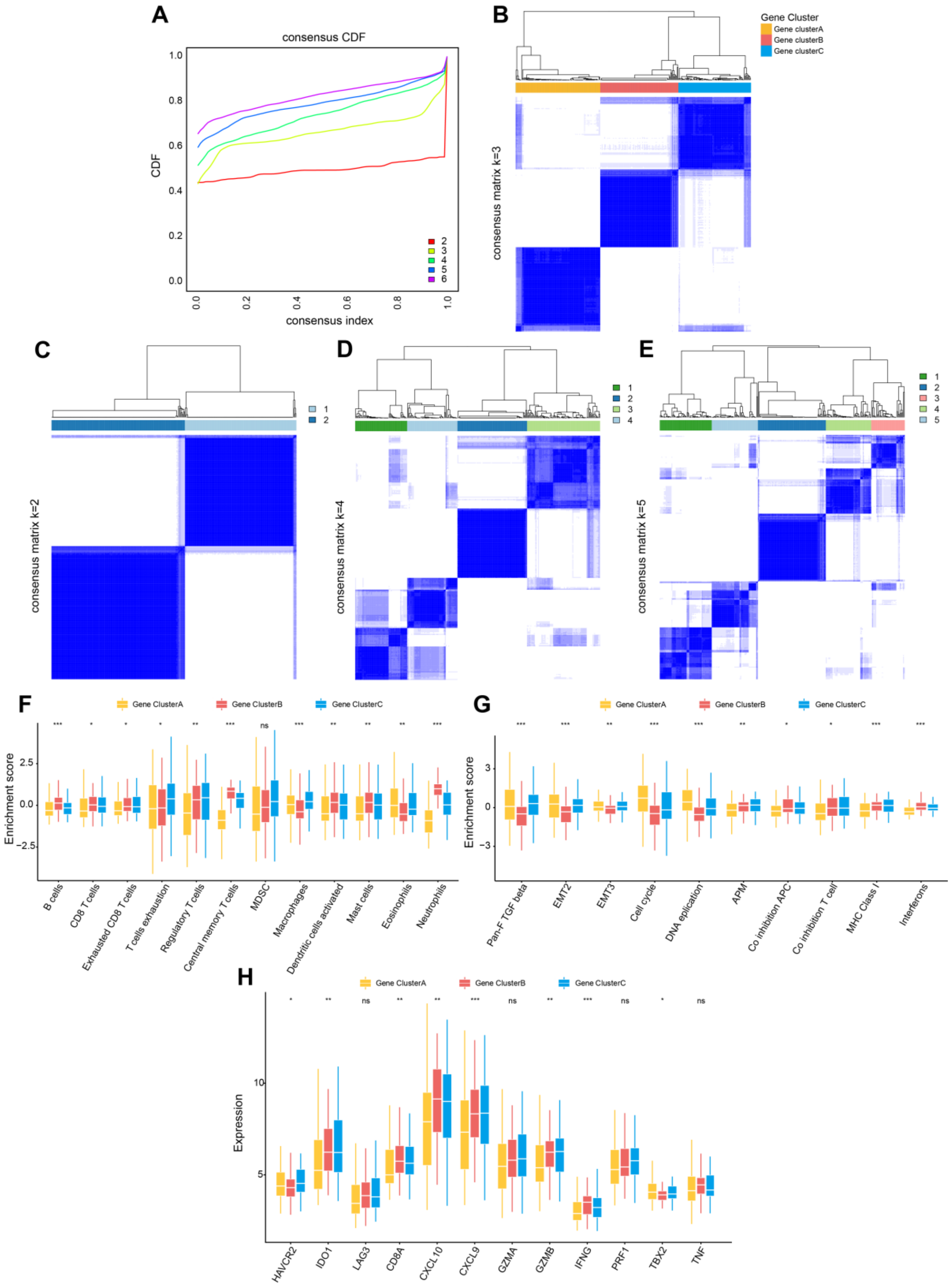

Figure S2 Characteristics of immune-cell infiltration, TME signature, immune checkpoint, and chemokine transcriptomes of distinct gene clusters

(A–E) Consensus matrices of the TCGA-OSCC data (k = 2–5). **(F)** The enrichment scores of immune-cell infiltrations in three ferroptosis gene clusters. **(G)** The enrichment score of the TME signature of three ferroptosis gene clusters. **(H)** Immune-checkpoint-relevant genes and immune-activation-relevant genes expressed in three ferroptosis gene clusters. Significant differences among of three ferroptosis clusters were assessed using the Kruskal–Wallis test (ns: not significant, *P < 0.05, **P < 0.01, ***P < 0.001).

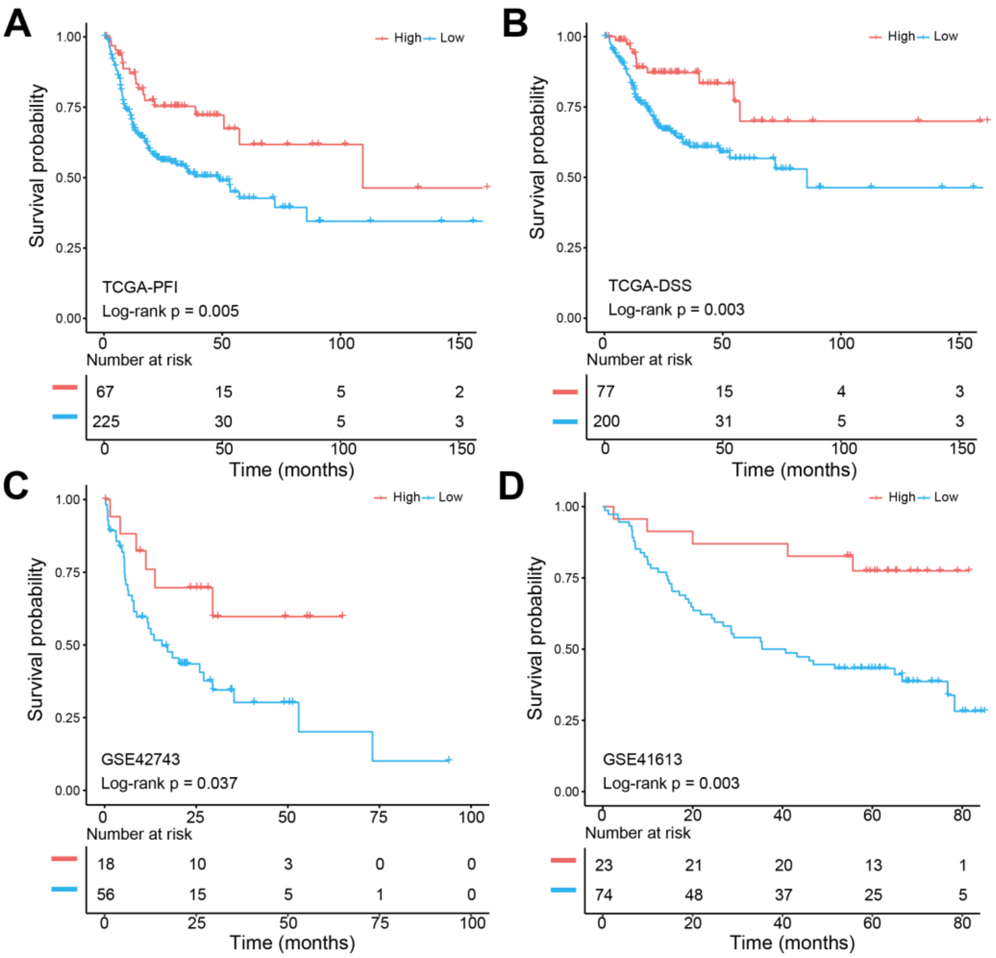

Figure S3 Significance of the FPscore in determining the roles of the outcomes

(A) Kaplan–Meier PFI curves for high and low FPscore subtype in the TCGA-OSCC cohort (log-rank test, P = 0.005). **(B)** Kaplan–Meier analysis of DSS of the high and low FPscore subtypes in the TCGA-OSCC cohort (log-rank test, P = 0.003). **(C, D)** Kaplan–Meier analysis of the OS of high and low FPscore subtype in the GES42743 cohort (log-rank test, P = 0.037) and GSE41613 cohort (log-rank test, P = 0.003).

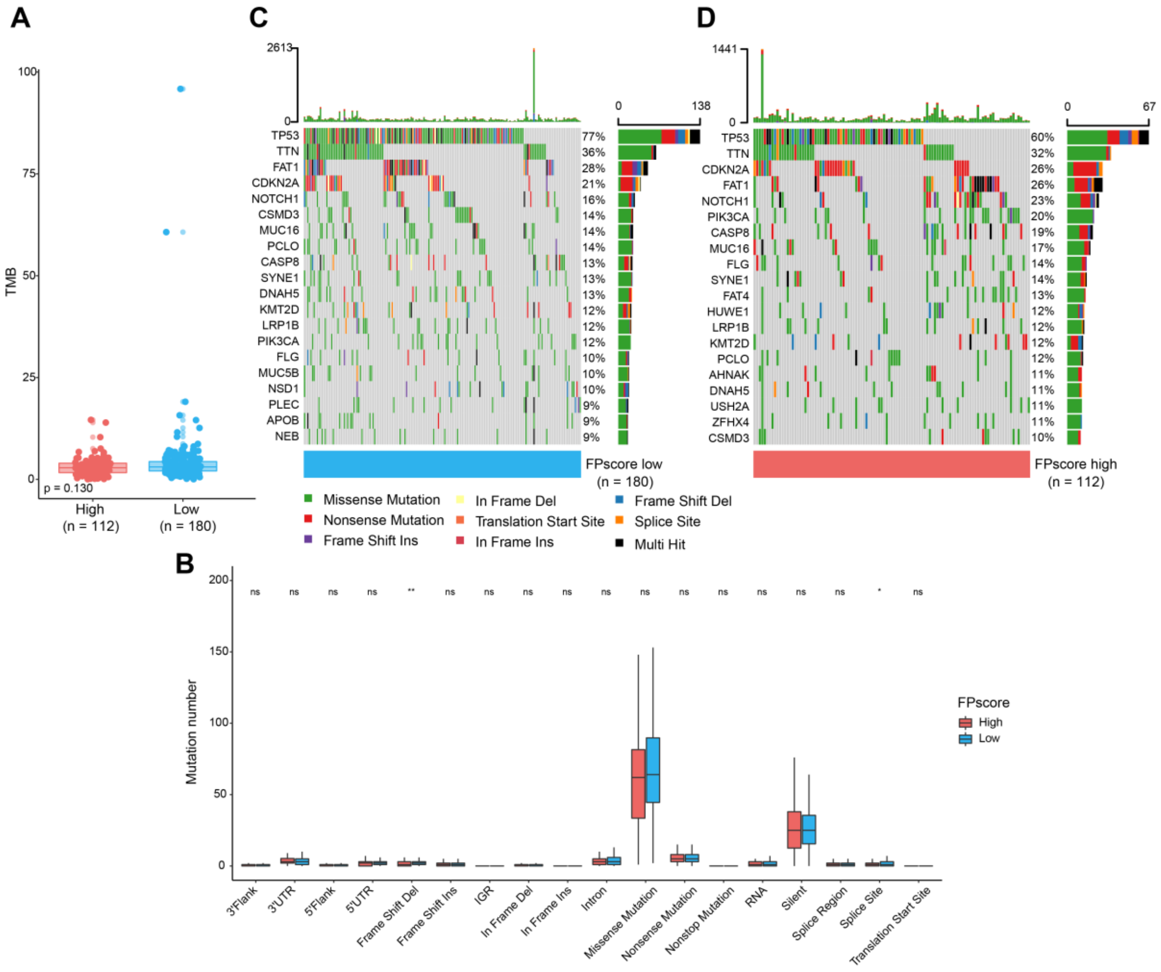

Figure S4 Correlation between the FPscore subtypes and somatic mutations

(A) The distributions of TMB differences in the high and low FPscore subtype (P = 0.13). **(B)** Boxplots showing the number of each mutation type in FPscore subtypes. **(C, D)** Mutational landscape of the top 20 most frequent mutated genes in the TCGA-OSCC cohort stratified according to low (blue bar) and high FPscore (red bar) subtype. Individual patients are represented in each column. The upper bar plot shows mutation number, the right bar plot shows the mutation frequency of each gene in the separate FPscore subtypes. Mutational signatures are shown as patient annotations. P values were tested using the Mann–Whitney test. (ns: not significant, *P < 0.05, **P < 0.01, ***P < 0.001).

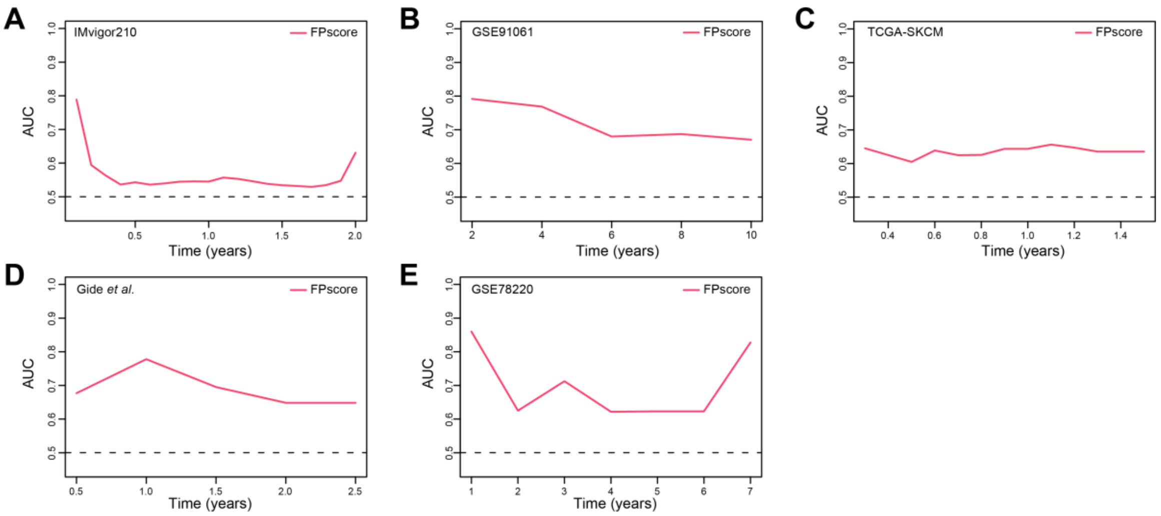

Figure S5 Time-dependent ROC curve of the immunotherapy cohorts

(A–E) Time-dependent ROC curves for predicting OS of the independent immunotherapy cohort, including IMvigor210, GSE910161, TCGA-SKCM, Gide et al. cohort, and GSE78220.
